# Doppler evaluation of renal resistivity index in healthy conscious horses and donkeys

**DOI:** 10.1371/journal.pone.0228741

**Published:** 2020-02-06

**Authors:** Francesca Freccero, Marina Petrucelli, Mario Cipone, Irene Nocera, Micaela Sgorbini

**Affiliations:** 1 Department of Veterinary Medical Sciences, Ozzano dell’Emilia, Bologna, Italy; 2 School of Biosciences and Veterinary Medicine, Veterinary Teaching Hospital, Matelica, MC, Italy; 3 Department of Veterinary Sciences, Veterinary Teaching Hospital “Mario Modenato”, San Piero a Grado, PI, Italy; Tokyo Joshi Ika Daigaku Toyo Igaku Kenkyujo Clinic, JAPAN

## Abstract

The renal resistive index (RRI) is used as a measurement of downstream resistance in arteries. The aim of this study was to assess the RRI of the arcuate arteries by pulsed-wave Doppler ultrasonography in healthy conscious horses and donkeys, and to verify any differences related to age, breed, bodyweight (BW) or body condition (BCS). Thirty-three healthy conscious horses and nine donkeys had their systolic and diastolic flow velocities at the level of the arcuate arteries estimated by pulsed-wave Doppler ultrasound, and the RRI was calculated. The relationship of RRI with age, breed (Trotters vs. other breeds), bodyweight (BW), and body condition score (BCS) were evaluated. PW Doppler evaluation of RRI was successfully applied in most of the horses, but to date not in the donkeys. In horses, median RRI values for the right kidney (0.58±0.006) were statistically higher than for the left (0.51±0.006). For the donkeys the values were comparable. There was no significant difference in RRI between horses younger or older than 15 years, and between breeds in horses. No correlation with age, BW or BCS was found in either horses or donkeys.

## Introduction

In human medicine, it has been long considered a good tool to evaluate renal microcirculation in both physiological and pathological conditions [1], by means of investigating arterial compliance and/or resistance [2]. However, RRI is affected by a complex interplay of renal and extra-renal factors (i.e. arterial and systemic hemodynamic factors). Thus, the interpretation of RRI as solely reflective of renal vascular resistance has to be made in consideration of the systemic hemodynamic and cardiovascular status [1].

When vascular resistance increases due to obstruction or vasoconstriction, diastolic blood flow slows down with respect to systolic blood [3]. This produces a higher decrease in end-diastolic velocity rather than in peak systolic velocity and, therefore increases RRI [4].

Using the RRI to assess the resistance at distal arterial branches (arcuate or interlobar) reveals the degree of intrarenal damage and can also be used to evaluate renal transplants in humans [5] and small animals [6,7,8].

There are few papers on the application of RRI in equine medicine. Hoffmann et al. (1997) [9] recorded Doppler-derived systolic and diastolic renal blood flow velocities and RI at the level of arcuate arteries in horses tranquilized with acepromazine. Macrì et al. (2015) [10] reported a difference in RRI between the right and the left kidney in conscious horses.

Both studies included a small number of animals. To the best of our knowledge, no studies have evaluated the RRI by Doppler ultrasonography in donkeys.

The aim of this study was to determine the RRI of the arcuate arteries by pulsed-wave Doppler ultrasonography in conscious healthy horses and donkeys. The relationship with age, breed, bodyweight and body condition was also investigated.

## Materials and methods

This study was approved by the Ethical Committee (University of Pisa) (Prot. N. 29913/18). Enrolled animals were not athletes but were employed in breeding programs at the Department of Veterinary Sciences, University of Pisa and at the Italian National Institute of Artificial Insemination (INFA), Department of Veterinary Medical Sciences, University of Bologna. Animals were housed in paddocks and had access to water ad libitum.

The inclusion criteria were: 1) no history of renal diseases; 2) normal physical examination with specific regard to cardiovascular system, serum urea and creatinine concentrations within normal limits, and no renal abnormalities detected by ultrasonography; 3) arterial blood pressure measured noninvasively before ultrasonography within normal ranges for equine species (135/90 mmHg, systolic/diastolic arterial pressure) [11].

Age, body weight (BW) and body condition score (BCS) [12] were recorded both for horses and donkeys. Each animal underwent trans-abdominal renal ultrasound once. Animals were not sedated, but just restrained in a handling box where they had already been acclimated. They were prepared by clipping an area over the 15th-17th intercostal spaces and the paralumbar fossa on the left side, and over the 14th-17th intercostal spaces and the paralumbar fossa on the right side [9]. Alcohol coupled with ultrasound gel was applied.

Ultrasonography of the right and left kidney was performed in a random order in different animals. A single operator with ultrasonography technical skills used a portable machine (MyLab30 Gold, Esaote, Italy) equipped with a multifrequency curvilinear probe 3.5–5 MHz. B-mode ultrasound was performed at a frequency of 3.5 MHz, depth and gain settings were changed as needed to optimize image quality. The focus was set at the level of the area of interest. Doppler ultrasound was performed at a frequency of 2.0 MHz. The wall filter was set on 50 MHz. The pulse repetition frequency (PRF) was set at the lowest possible values that allowed the flow to be displayed without aliasing. The Doppler gain was adjusted to optimize the spectral displays. No beam-angle correction was employed.

To identify the optimal acoustic window for Doppler display, B-mode ultrasound images were obtained in the dorsal and transverse anatomic planes of the right and left kidneys [13]. Once the optimal B-mode image was visualized, the arcuate arteries were identified at the level of the corticomedullary junction by color Doppler. Pulsed wave (PW) Doppler modality was then activated and velocity flow examination was performed by placing the sample volume (size set at 2 mm) at the intraluminal level. For each kidney, at least one 8-second PW Doppler tracing was stored.

The PW Doppler spectra were analyzed offline by one observer, using MyLabDesk, (Esaote, Italy). Only Doppler flow waveforms with clear and complete velocity signals were measured. The peak systolic velocity (PSV) and end-diastolic velocity (EDV) of the same cardiac cycle were manually traced using the electronic caliper, and RRI values were automatically calculated using the formula: [(PSV-EDV)/PSV]. Three repeatable, but not necessarily consecutive, Doppler velocity waveforms were measured if available; tracings with two adequate velocity waveforms were also measured. Tracings with one waveform were excluded from statistical analysis. For each kidney, RRI values from two or three measurements were averaged. Data distribution was verified by the Kruskal-Wallis test. RRI values showed a non-Gaussian distribution, and were thus expressed as median and standard errors, with minimum and maximum values, for the right and left kidneys, both in horses and donkeys.

The Mann-Whitney U test was used to assess differences in RRI between the right and left kidney in the two species, and differences in RRI related to age (group Adult: horses <15 years old; group Old: horses ≥15 years old) [14] and breed (Trotters vs. other breeds) in horses. The Spearman test and the linear regression were performed to verify any correlations between RRI values and age, BW or BCS in the two species. Statistical significance was set at P<0.05. The statistical analysis was performed using GraphPad Prism 6 (GraphPad Software, La Jolla, California, USA).

## Results

The study included 42 animals: 33/42 horses and 9/42 donkeys. A total of 4/33 horses were stallions and 29/33 mares; 23/33 were Trotters and 10/33 other breeds (Thoroughbred, Warmblood).

The horses’ age ranged from 3–24 years (median 13 years). The groups ‘Adult’ and ‘Old’ included 18/33 (55%) and 15/33 (45%) animals, respectively. The BW was 370–645 kg (median 522 kg) and the BCS 4-6/9 (median 5/9).

All the donkeys were female of the Amiata-breed. Donkeys’ ages ranged from 7–13 years (median 10 years). The BW was 300–407 kg (median 358 kg), and the BCS of 5.5-7/9 (median 5.5/9).

Overall, the procedure was well tolerated. Mean time to satisfactory completion of the examination was usually within 60 minutes per animal. Doppler examination of the arcuate arteries was mostly performed bilaterally in the transverse or transverse-oblique anatomic plane in both horses and donkeys.

In horses, adequate PW Doppler tracings of the right kidney were obtained in 31/33 (94%) horses and of the left kidney in 32/33 (97%). Two or more Doppler waveforms were obtained in 24/31 (77.4%) and 27/32 (84.4%) for the right and left kidney, respectively.

The RRIs for the horses’ population, and in the subgroups ‘Adult’ vs. ‘Old’ and ‘Trotters’ vs. ‘other breeds’ are reported in Table 1.

**Table 1:** Renal resistivity index at the level of the arcuate arteries obtained by PW Doppler for the right and left kidneys in a population of conscious healthy horses, and in groups divided by age (younger vs. older than 15 years) and breed (Trotters vs. others).

Overall horses	Right kidney (n = 24)		Left kidney (n = 27)	
**Median±SE**	0.58±0.006*		0.51±0.006*	
**Max**	0.66		0.64	
**Min**	0.53		0.49	
**Age groups**	**ADULT (<15 years)**		**OLD (≥15 years)**	
	**Right kidney (n = 14)**	**Left kidney (n = 15)**	**Right kidney (n = 10)**	**Left kidney (n = 12)**
**Median±SE**	0.58±0.004	0.51±0.01	0.59±0.01	0.51±0.004
**Max**	0.59	0.49	0.62	0.52
**Min**	0.55	0.55	0.53	0.50
**Breed groups**	**TROTTERS**		**OTHER BREEDS**	
	**Right kidney (n = 14)**	**Left kidney (n = 18)**	**Right kidney (n = 10)**	**Left kidney (n = 9)**
**Median±SE**	0.58±0.01	0.51±0.01	0.59±0.01	0.51±0.003
**Max**	0.66	0.64	0.62	0.53
**Min**	0.53	0.49	0.55	0.50

^a^ SE: Standard Error; Max: maximum value; min: minimum value; n: number of horses included.

^b^ *: mean statistical difference in the same row (P<0.05).

**Fig 1:**
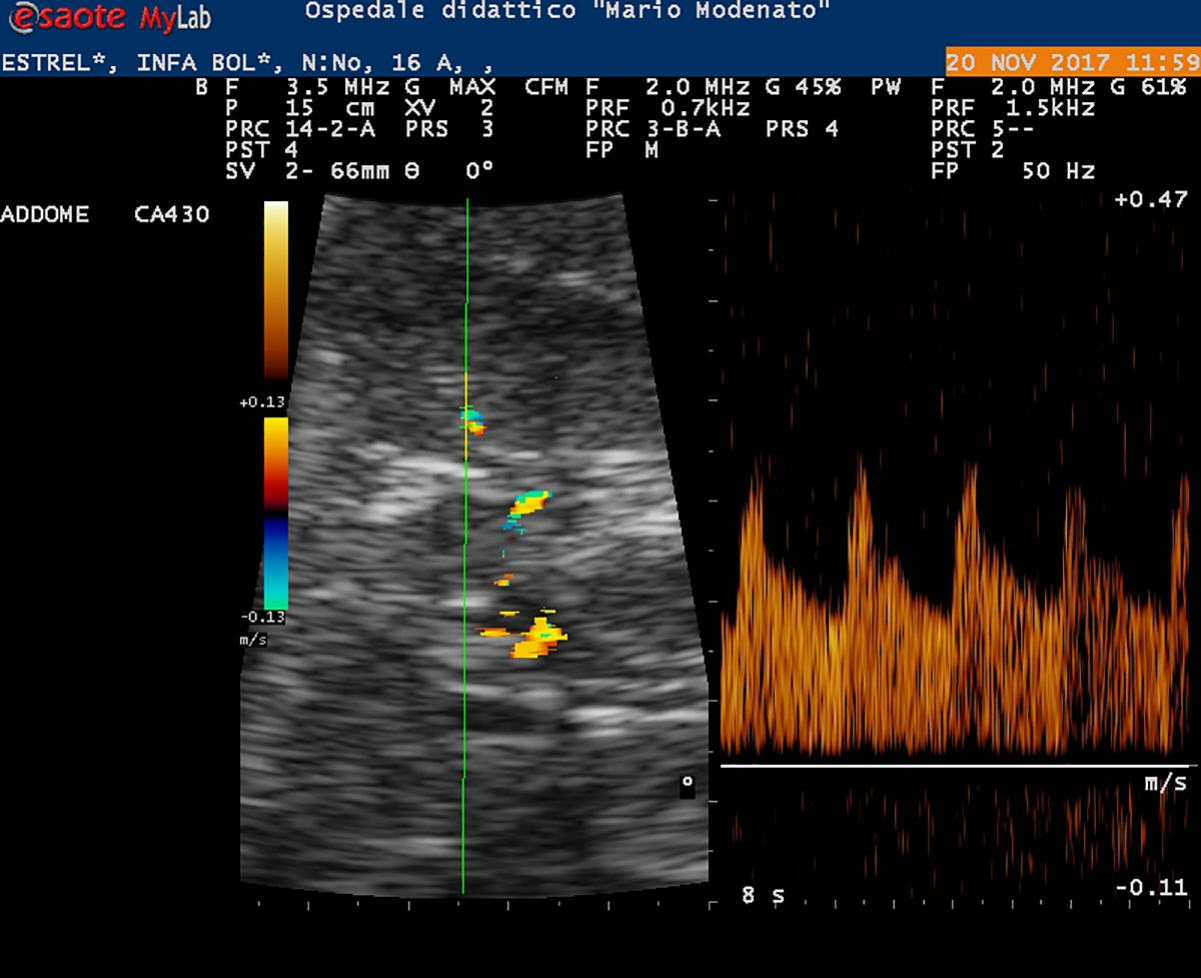
Doppler ultrasound image showing an intrarenal arterial Doppler spectrum of the kidney in a healthy conscious horse. The color Doppler superimposed on the B-mode image was used to identify an arcuate artery, and the PW Doppler sample volume was placed at the intraluminal level (left side of the image). Normal intrarenal Doppler flow velocity waveforms corresponding to four consecutive cardiac cycles (right side of the image).

In Fig 1, Doppler ultrasound image showing an intrarenal arterial PW Doppler spectrum of the kidney in a healthy conscious horse was visualized.

Statistical differences in RRI values between the right and the left kidney were obtained in the overall population (P<0.0001).

In donkeys, adequate PW Doppler tracings were obtained for both the right and left kidneys in 5/9 (55.5%) subjects. Two or more Doppler waveforms were obtained in 4/5 (80%) donkeys both the right and left kidney. The RRI values for donkeys are reported in Table 2.

**Table 2:** Renal resistivity index at the level of the arcuate arteries obtained by PW Doppler for the right and left kidneys in a group of conscious healthy donkeys.

Donkey group	Right kidney (n = 4)	Left kidney (n = 4)
**Median±ES**	0.58±0.02	0.53±0.01
**Max**	0.59	0.58
**min**	0.56	0.52

^a^ SE: Standard Error; Max: maximum value; min: minimum value; n: number of donkeys included.

No correlation was observed between age, BW or BCS and RRI nor for horses, neither for donkeys.

## Discussion

In this study, PW Doppler was successfully applied to estimate the RRI at the level of the arcuate arteries in a population of healthy conscious horses and donkeys. While the technique has been previously described in small samples of horses [9,10], to the best of our knowledge there are no reports on its use in donkeys.

In our study, a curvilinear probe working at 3.5 MHz was chosen to provide the highest resolution relative to the organs’ depth, together with color Doppler to help locate vessels, with the aim to sample RRI at the level of the arcuate of interlobar arteries, as it recommended in human and small animals [15,16].

In this study, kidneys were qualitatively assessed in terms of size, echotexture and echogenicity for enrollment, and fulfilled normal sonographic morphometric features previously described [13,17,18]. In most cases the optimal view for Doppler assessment was bilaterally the transverse or transverse-oblique plane. This partly differs from Hoffmann et al. (1997) [9], who used the sagittal and transverse plane in the right kidney and the dorsal plane in the left kidney to assess the arcuate arteries. This may suggest that there is no standard view but rather the optimal one to this aim might be largely dependent on individual animals. Irrespectively of the sonographic plane, once the arcuate vessels had been identified at the corticomedullary junction as described [9], the PW Doppler angle was always optimized with respect to the long axis of the artery (<60°) to obtain a reliable velocity measurement.

Adequate PW Doppler tracings were successfully obtained for almost all the kidneys in the horses. The technique requires meticulousness, some manual experience and may take time to acquire satisfactory data, mainly because of motion and other (e.g. gas content, breathing) unavoidable causes of artifacts and Doppler signal unsteadiness. However, compared to previous approaches [9], recent technological progress has facilitated the use also in non-sedated horses.

The technique also worked in donkeys though it was less effective in obtaining qualitatively adequate tracings for RRI estimation in this group.

Less cooperativity and/or specific differences (i.e. conformation, thickness of the skin and subcutaneous tissue, acoustic window) may also have contributed to the technique not working as well with donkeys as in horses. There are inter-specific dissimilarities in subcutaneous fat distribution: compared to horses, donkeys have higher accumulations of adipose tissue in the abdominal wall and other body sites [19]. This poses more technical difficulties in the optimal imaging of the kidney using the transabdominal approach [20], as well as producing higher ultrasound attenuation [19].

There are also fewer arcuate arteries in donkeys with respect to horses, thus adequate spectra might be more challenging to acquire [21].

The RRIs at the arcuate arteries in our study are similar to values reported by others [9] in acepromazine-tranquilized horses (0.549 +/- 0.044), and in a small group of conscious horses [10]. Although the authors suggest acepromazine might decrease systemic vascular resistance and blood pressure [9], similarly to horses, the RRIs measured in conscious and sedated cats were comparable to each other [8].

Renal RI values in the present and previous studies in horses [9,10] fall in the lower reference ranges for small animals and humans, where about 0.7 is considered the upper limit of normality in adults [5,15].

Our results confirm that in horses, the RRI in the right kidney is statistically higher than in the left kidney [10]. To the best of our knowledge, in other species there have been no consistent differences in RI reported between the right and left kidney in physiological conditions.

Doppler investigation of right vs. left kidney had no consistently significant effect on RI in humans, but very occasionally on other Doppler parameters [22,23]. Measurement of intrarenal RI in the right and left kidney did not influence the results in healthy dogs and cats in earlier studies [15,24], while Tipisca et al. (2016) [8] reported significantly higher RI in the left vs. the right kidney in healthy cats.

The difference in RRI between kidneys is difficult to explain. Unlike other species, anatomical differences between the right and left kidney in equines are marked [21,25]. However, using a stereological method the kidneys showed similar weight, density, volume and volume fractions (cortex, medulla, pelvis). Whether there are any microscopical differences in functional subunits is not known [26].

The right and left renal arteries merge at slightly different levels and angulation with respect to the aorta [25], and the branching and distribution of the principal renal vessels is not completely symmetric between sides [9]. Vasculature geometry has effects on blood hemodynamics [27], which this might be speculated to affect vascular resistivity.

Furthermore, there are no proven different physiological and/or hemodynamic features between the kidneys. As far as we know, only one study has shown ‘a significant difference in the split function of the equine kidneys, with the glomerular filtration rate (GFR) of the right kidney contributing 60.1 +/- 9.12% of the total function, as determined by scintigraphy [29]. The GFR is a measure of functional renal mass [27], but notably GFR and blood flow, and so the RI, are not comparable measures of renal function [28] and they cannot be directly linked at this point. In humans, asymmetry in renal blood flow has been reported between the kidneys in normal subjects and in hypertensive patients, with no convincing anatomic-physiological explanation [23].

Beside the fact that ultrasonography is essentially an operator-dependent method, many other technical factors, such as the different depths of the kidneys, the sonographic window (e.g. acoustic interfaces, fat), and Doppler angle, all potentially affect Doppler estimation of both PSV and EDV equally and thus should not affect the value of derived index (RI).

There are no reports of renal Doppler assessment in donkeys. In our study, the RRIs in donkeys were similar and within the ranges obtained in horses.

In contrast, we found no differences between the right and left kidney in donkeys. Besides species-specific anatomical and/or technical factors possibly affecting RRI, as previously accounted, we believe that we detected no statistical significance probably due to the small size of the sample. However, the existence of a difference in renal hemodynamic between horses and donkeys cannot be ruled out, given that donkeys have less arcuate arteries [21]. On the other hand, no differences were found in GFR between adult horses and donkeys in one study [29]. Interestingly, in our study the age of the horses had no effect on RRI either in horses or donkeys. In contrast, aging has a positive statistically significant effect on RRI in humans, particularly those over 50 years of age [22,30], but inconsistently in small animals [8,31].

As in small animals, chronic renal failure (CRF) seems more represented in older horses, with the prevalence increasing to 0.23% in horses older than 15 years and is 0.51% for intact males over 15 years of age [32].

However, it is possible that our finding is affected by the composition and dimension of the sample.

No significant differences in RRI were found comparing Trotters to other breeds. No normal ranges of RRI have been reported for Standardbreds, however our results are comparable to values obtained in conscious Thoroughbred horses [10]. Although we only considered light breeds, we think that significant breed-related differences would not be expected. To our knowledge, there are no data on equine breed-related differences in kidney function. Similarly, despite considerable variations in size, no differences in RRI related to breed have been reported in dogs and cats [31].

A few studies have investigated the RRI and weight relationship in veterinary species. Park et al. (2008) [33] observed a weak correlation in cats, while Ostrowska et al. (2016) [31] found no correlation between BW and RRI in dogs and cats.

Similarly to Ostrowska et al. (2016) [31], we found no correlation between RRI and BW either in horses or donkeys, and similarly between RRI and BCS.

As in other animals, in horses body fat is the most variable body tissue, and it increases with BW and BCS [34]. Interestingly, in humans, weight excess and/or a central body fat distribution are associated with an unfavorable renal hemodynamic profile, which may play a role in chronic renal damage [35] and is characterized by higher intra-renal resistances (i.e. RI) [36].

The relationship between body fat distribution and intra-renal resistance in equines demand further investigations.

One limitation is that we did not perform a technique or measurements variability study. This has not been investigated in equines, but the evaluation of intrarenal blood flow with Doppler indices such as RI has been described as an accurate and reliable technique in humans [37], and no significant inter- or intra-observer variation occurred in measuring the RI in humans [38].

In conclusion, PW Doppler evaluation of RRI in horses and donkeys is feasible but time-consuming and requires technical ultrasonographic skills. The median RRI values obtained for the right and left kidney in horses in a larger population of subjects confirmed the differences previously reported. Further studies are needed to clarify the relationship between RRI and morpho-functional features of the kidneys, age and BW or BCS in horses.

In donkeys, RRI values are comparable to horses. However, to verify similarities or dissimilarities between the two species a larger population needs to be studied.
